# Assessing Paramedics’ Competence and Training in End-of-Life Care: A Cross-Sectional Study in Saudi Arabia

**DOI:** 10.3390/clinpract15030046

**Published:** 2025-02-25

**Authors:** Ahmed Alanazy, Fatimah Khalifah Alsahli, Zahra Essam Alhassan, Zahra Hassan Alabdrabulridha, Moneerah Khalifah Aljomaan, Abdullah Alruwaili

**Affiliations:** 1 Emergency Medical Services Program, College of Applied Medical Sciences, King Saud Bin Abdulaziz University for Health Sciences, Al Ahsa 31982, Saudi Arabia; 2 King Abdullah International Medical Research Center, Al Ahsa 31982, Saudi Arabia; 3 Ministry of National Guard-Health Affairs, Al Ahsa 31982, Saudi Arabia

**Keywords:** end-of-life care, paramedics, palliative care, training, Saudi Arabia, cross-sectional study

## Abstract

**Background**: End-of-life (EOL) care is an integral part of paramedic services, requiring not only medical expertise but also communication skills and emotional support. With the evolving role of paramedics in providing palliative care, understanding their attitudes toward EOL care and the impact of specialized training becomes crucial. **Aim**: This study aims to assess the attitudes of Saudi Arabian paramedics toward EOL care and evaluate the influence of prior EOL care training on these attitudes. **Methods**: A cross-sectional study was conducted among paramedics in Saudi Arabia using convenience and snowball sampling. Data were collected via an online survey distributed through emails and social networks, encompassing demographic information and attitudes toward EOL care. The survey was structured into two parts, with the second part developed from the relevant literature. Statistical analysis was performed using STATA version 18, employing chi-squared and Fischer exact tests for comparison. **Results**: The study involved 1049 paramedics, with the majority being aged 26–35 years (54.43%) and predominantly male (65.59%). About half of the participants (50.43%) had previously participated in EOL care courses. Paramedics who received EOL training demonstrated significantly more positive attitudes toward the role of EOL care in their jobs (98.49% versus 32.12%, *p* < 0.001) and were more comfortable discussing death with patients (51.42% versus 29.23%, *p* < 0.001). A significant majority viewed caring for a dying patient as a worthwhile experience (95.42%), and 95.33% agreed on the importance of involving the patient’s family in care. **Conclusions**: The findings highlight the positive impact of EOL care training on paramedics’ attitudes toward palliative care. Specialized training enhances paramedics’ comfort in discussing death and their perceptions of the role of EOL care, underscoring the need for integrating comprehensive palliative care education into paramedic training programs. Future research should focus on developing standardized EOL care courses to further explore their impact on paramedics’ knowledge, attitudes, and practices.

## 1. Introduction

The global aging population has brought to light a significant gap in the preparedness of healthcare professionals to adequately address the needs of older adults, including care at the end of life (EOL). Studies have shown that university programs often lack sufficient content on aging and active aging, leaving healthcare providers, including paramedics, inadequately equipped to address the complex needs of this demographic [1].

EOL care represents a critical component of healthcare, aimed at providing comfort, dignity, and pain relief to individuals in the final stages of life [2]. The importance of this care cannot be overstated, given its profound impact on the quality of life for patients and their families [3]. Despite its significance, EOL care presents substantial challenges, not least because of the emotional and logistical complexities involved [4]. As healthcare systems worldwide strive to meet these challenges, the role of paramedics in the EOL care process has increasingly come into focus.

Paramedics, often the first point of contact for patients accessing emergency medical services, are uniquely positioned to significantly influence the EOL care trajectory [5]. Their involvement in EOL care extends beyond mere crisis intervention to encompass a comprehensive approach which includes pain management, emotional support, and the facilitation of hospital-to-home transitions [6]. This expanded role underscores the need for a deeper understanding of paramedics’ experiences, competencies, and challenges faced in delivering EOL care.

The challenges paramedics encounter in EOL situations are multifaceted [7]. They range from the emotional toll of caring for dying patients and their families to practical difficulties in decision making and communication. Furthermore, paramedics often navigate these challenges without the extensive palliative care training which might prepare them for the nuanced demands of EOL care. These issues are compounded by the high-pressure, time-sensitive nature of emergency medical services, highlighting a significant gap in the literature and practice concerning paramedics’ preparedness and support systems for EOL care [8].

Existing research has predominantly focused on the clinical and technical aspects of paramedics’ roles, with less attention given to their involvement in EOL care. This oversight represents a critical gap in our understanding of the emergency care continuum, particularly in addressing the needs of patients at the end of life. Recognizing this gap, our research aims to explore the attitudes of paramedics toward EOL care, assess the impact of prior EOL care training on these attitudes, and identify the challenges paramedics face in this context. In the process, we seek to contribute valuable insights into enhancing the quality of EOL care provided by paramedics, informing both practice and policy to better support paramedics and the patients they serve.

## 2. Materials and Methods

### 2.1. Study Design and Period

This cross-sectional study was conducted to assess the attitudes of Saudi Arabian paramedics toward EOL care and examine the influence of prior EOL care training on these attitudes.

### 2.2. Ethical Considerations

Ethical approval was obtained from the Institutional Review Board (IRB) at the King Abdullah International Medical Research Center (KAIMRC) under approval number SPA24/016/9. Prior to participation, all participants were informed about the study’s objectives and procedures. Informed consent was acquired electronically, requiring participants to sign a consent form to acknowledge their informed and voluntary participation before accessing the survey.

### 2.3. Population and Sampling

This study targeted paramedics working in Saudi Arabia. To achieve a representative sample size, a combination of convenience sampling and snowball sampling methods was employed. This dual approach allowed for the initial recruitment of participants through direct contacts and subsequently expanded the sample through referrals within the paramedics’ professional and social networks. The survey link was disseminated via emails through professional networks and social media platforms (WhatsApp groups, X, and Instagram), facilitating broad participation. This study welcomed participants regardless of their age, gender, years of experience, or location of work.

### 2.4. Data Collection

Data were collected through a structured online survey distributed via Google Docs consisting of two sections. The first section gathered demographic and professional information, including age, gender, marital status, educational level, job role (EMT or paramedic), work setting, geographical area of work, years of experience, daily working hours, and the number of occasions dealing with EOL patients. The second section assessed paramedics’ attitudes toward EOL care, developed based on a thorough literature review and elements adapted from two previous studies [9,10]. This section included 16 questions divided into four practice-related items answered with “yes” or “no” and 12 attitude-related items focusing on care provision to EOL patients, with responses recorded as “agree”, “disagree”, or “unsure”. Given that prior studies used a five-point Likert scale (from strongly disagree to strongly agree), this study modified the response format to a three-option scale to enhance clarity and encourage higher response rates among paramedics. Additionally, while some questions were adapted from previous research, new questions were developed to address the unique role of paramedics in EOL care, particularly in emergency and pre-hospital settings. This adaptation ensured relevance to paramedic practice while maintaining comparability with prior research. The survey items are detailed in this study’s tables, with frequencies replacing numerical counts for clearer interpretation in Tables. To ensure the reliability of the survey, the second section was validated using Cronbach’s alpha, achieving a score of 0.85, which is above the generally accepted threshold of 0.7, indicating good internal consistency. This validation supports the credibility of the collected data and the robustness of this study’s findings.

### 2.5. Sample Size Calculation

The sample size was calculated to accurately estimate the proportion of paramedics with positive attitudes toward EOL care. Based on a presumed proportion of 50%, a 5% margin of error, and a 95% confidence level, the calculation accounted for the minimum sample size needed of 315 responses. An additional adjustment was made to compensate for an anticipated 12.5% non-response rate based on our previous research [11], ensuring the study’s statistical precision in representing the target population.

### 2.6. Statistical Analysis

Data analysis was performed using STATA version 18. The demographic characteristics and attitudes toward EOL care were summarized using descriptive statistics (numbers and percentages). To compare the attitudes between paramedics who had received prior EOL care courses and those who had not, chi-squared tests were primarily used. Fischer exact tests were applied in instances where the number of responses in any category was below five, ensuring the statistical reliability of the comparison.

## 3. Results

### 3.1. Participant Demographics and Professional Backgrounds

The participant pool was predominantly in the age group of 26–35 years (54.43%), with a significant majority being male (65.59%) versus female (34.41%). These paramedics were almost equally divided between those working in the private sector (55.48%) and those in government positions (44.52%), highlighting the diversity of the employment settings. Their experiences spanned across various years, with the largest group having 6–10 years of experience (34.99%), followed closely by those with 11–15 years of experience (25.64%). The working hours varied significantly, with nearly half (49.57%) working 12 h shifts and a quarter (25.17%) engaged in 24 h shifts, underscoring the demanding nature of their roles. This demographic also included a balanced geographical representation from both rural (55.29%) and urban (44.71%) areas. Most respondents were employed in hospital-based settings (58.91%), held a bachelor’s degree (97.81%), and had substantial experience dealing with EOL patients, with 54.72% having encountered such situations in their line of duty (Table 1).

### 3.2. Attitudes Toward End-of-Life Care

The findings indicated strong recognition among paramedics of the importance of EOL care as part of their professional responsibilities, with 65.59% affirming its centrality to their role. However, exposure to palliative care education through seminars or lectures was relatively divided, with 45.19% reporting attendance. Notably, exactly half of the respondents (50.43%) had participated in EOL care courses. The inclusion of EOL care in undergraduate curricula was almost universal among the participants (97.14%).

Attitudes toward specific aspects of EOL care were generally positive. A significant majority saw the value in EOL care, with 95.42% agreeing that caring for a dying patient is a worthwhile experience. Additionally, the necessity of involving the patient’s family in care was overwhelmingly supported (95.33%). Moreover, an overwhelming majority expressed a willingness to engage in EOL care (95.81%), demonstrating a strong commitment to this aspect of patient care. Despite this, challenges were evident, as 57.29% of the paramedics felt uncomfortable discussing impending death with patients, and 57.96% were frustrated by the time commitment required in providing EOL care. Additionally, opinions were divided on the matter of honesty with patients about their conditions, with 42.8% agreeing that patients should be given honest answers and a considerable portion remaining unsure (55%), reflecting the complexity of communication in EOL situations (Table 2).

### 3.3. Impact of End-of-Life Care Courses

The impact of prior EOL care courses was evident across several dimensions. Paramedics who had received EOL courses demonstrated significantly more positive attitudes toward the role of EOL care in their job, with 98.49% acknowledging it as playing a key role compared with only 32.12% among those without such education (*p* < 0.001). Additionally, these courses improved comfort in discussing death with patients (51.42% versus 29.23%, *p* < 0.001), suggesting that education can alleviate some of the emotional challenges associated with EOL care (Table 3).

Interestingly, the study also noted a correlation between the receipt of EOL courses and the participants’ years of experience and daily working hours. Trained paramedics were found across a broader spectrum of experience levels and were more likely to work longer shifts, indicating that those with more extensive EOL training may be more engaged in their professional roles.

Family involvement in EOL care and honesty with patients about their conditions were also influenced by EOL training. While a slightly lower percentage of trained paramedics advocated for family involvement compared with their untrained peers (92.44% versus 98.27%, *p* < 0.001), they showed a greater inclination toward honesty with EOL patients (54.82% agree versus 30.58%, *p* < 0.001), highlighting the nuanced understanding and empathy developed through EOL education (Table 4).

## 4. Discussion

This study explored paramedics’ attitudes toward EOL care and assessed the impact of EOL care courses on these attitudes. The findings offer significant insights into the complexities of providing EOL care in emergency medical services and underscore the value of specialized training in palliative care. Paramedics who received EOL training were more likely to recognize EOL care as essential to their role (98.49% versus 32.12%, *p* < 0.001) and felt more comfortable discussing death with patients (51.42% versus 29.23%, *p* < 0.001). The majority viewed EOL care as meaningful (95.42%) and supported family involvement (95.33%), yet challenges remained, with 57.29% feeling uncomfortable discussing death and 57.96% frustrated by the time demands. Additionally, trained paramedics were more likely to advocate for patient honesty (54.82% versus 30.58%, *p* < 0.001) and were engaged in roles requiring EOL care. These findings highlight the need for structured and standardized EOL training to enhance paramedics’ confidence, communication skills, and overall ability to provide compassionate end-of-life care.

The demographic data revealed a young and predominantly male participant pool, reflecting broader trends in the paramedic profession. The almost equal split between private and government sectors, along with the distribution of experience and working hours, underscores the diversity within the emergency medical services profession. Importantly, the majority of the paramedics had encountered EOL patients in their line of duty, emphasizing the relevance of EOL care training in their professional practice.

The strong affirmation (65.59%) of EOL care as a central role for paramedics indicates a recognition of the importance of palliative care within emergency medical services. This is particularly noteworthy given the traditionally acute care focus of paramedic services. The findings that half of the respondents had participated in EOL care courses and almost all had EOL care included in their undergraduate curricula suggest a growing emphasis on palliative education within paramedic training programs. This recognition aligns with the broader body of research which highlights the evolving role of paramedics from solely acute care providers to integral parts of a comprehensive palliative care approach. Studies such as that by Juhrmann et al. [12] have documented the expanding scope of paramedic practice to include palliative and EOL care, echoing our findings on the paramedics’ perceived roles and responsibilities.

The positive attitudes toward EOL care, including the willingness to engage in such care and the value placed on family involvement, mirror the principles of palliative care outlined in the seminal work by Andershed et al. [13]. These principles emphasize a holistic approach to patient care, addressing not just physical symptoms but also psychological, social, and spiritual needs and underscoring the importance of family as part of the care team [14].

However, our findings also shed light on the challenges paramedics face, notably discomfort in discussing impending death and frustration with the time commitment required. This reflects the broader challenges within healthcare professions regarding EOL care, as identified in the literature. For instance, studies by Collins et al. [15] and Bergenholtz et al. [16] have highlighted the discomfort many healthcare providers feel when navigating conversations about death and dying, pointing to the need for improved communication training as part of EOL care education. The high value placed on EOL care and family involvement is consistent with the literature emphasizing the importance of holistic care at the end of life, which includes not only the physical but also the emotional, social, and spiritual needs of patients and their families [14]. The discomfort and frustration reported by paramedics echo the findings from other healthcare settings, where professionals often feel inadequately prepared to address the complex needs of EOL patients and their families.

The significant impact of EOL care courses on paramedics’ attitudes underscores the importance of targeted education and training. Similar research has shown that healthcare professionals who receive specialized palliative care training are more confident, comfortable, and competent in providing EOL care. The research by Carter et al. [17] demonstrated that healthcare professionals, including paramedics who received palliative care training, reported increased confidence and a better ability to provide compassionate and effective EOL care. This aligns with our observation that the paramedics with EOL training were significantly more positive about their roles in EOL care and more comfortable discussing death with patients. The correlation between the receipt of EOL courses and the paramedics’ years of experience and daily working hours suggests that those with more extensive training are more engaged and possibly more resilient in the face of the demands of EOL care.

The alignment of our findings with existing evidence reinforces the call for integrating comprehensive EOL care training into paramedic education and ongoing professional development. This integration should not only focus on the technical aspects of palliative care but also extensively cover communication skills, ethical considerations, emotional support, and family engagement strategies. An emerging area of interest which aligns with our findings is the role of dyadic research in understanding and improving EOL care. Dyadic research, which examines the interdependent relationships between patients and their caregivers (including family members and healthcare professionals) [18], offers valuable insights into the mutual influences of attitudes, perceptions, and experiences in the EOL care context. This approach can unravel the complex dynamics of EOL care interactions, emphasizing the multi-directional impact of emotions, behaviors, and communication between patients, caring families, and care givers [19]. By incorporating dyadic methodologies, future research can delve deeper into how paramedic attitudes toward EOL care affect patient and family experiences and vice versa, offering a holistic view of the care process.

Incorporating dyadic perspectives into EOL care research can significantly inform training programs for paramedics by highlighting the importance of empathetic communication, understanding patient and family needs, and recognizing the emotional labor involved in EOL care. Studies focusing on dyadic interactions in EOL situations can reveal effective strategies for managing the challenges identified in our study, such as discomfort in discussing impending death and the emotional toll of prolonged engagement with dying patients and their families.

## 5. Limitations, Literature Gaps, and Future Research Directions

While this study provides valuable insights, it is not without limitations. The self-reported nature of the data may have introduced response bias, and the demographic characteristics of the sample may have limited the generalizability of the findings. Additionally, analyses were not stratified according to the area of care, which could have provided a more nuanced understanding of how different work environments influence paramedics’ attitudes toward EOL care. A notable limitation is the lack of a well-defined parameter for prior EOL training courses. The respondents’ backgrounds varied significantly, meaning that the EOL care courses they attended differed in content, duration, and depth. This variation introduces uncertainty regarding which specific aspects of EOL care were covered in their training and how comprehensively these aspects were addressed. Consequently, this study may not have fully captured the potential benefits of a standardized, comprehensive EOL care curriculum. This issue is currently being explored further by our research department to assess how standardized training can enhance paramedics’ preparedness for EOL care.

While our findings contribute to the growing body of evidence on the importance of EOL care in paramedic practice, they also reveal gaps in the literature, particularly regarding the long-term impacts of EOL care training on patient and family outcomes in emergency settings. Future research should aim to address these gaps by investigating the direct effects of paramedic-provided EOL care on patient comfort, satisfaction, and the overall quality of life at the end of life. Additionally, further studies should explore the experiences and perceptions of families receiving paramedic-led EOL care, as well as strategies to enhance paramedics’ competencies in delivering compassionate and effective EOL care.

## 6. Conclusions

This study underscores the importance of EOL care in paramedic practice and the positive impact of specialized training on paramedics’ attitudes and confidence. Trained paramedics were more likely to recognize EOL care as essential (98.49% versus 32.12%, *p* < 0.001), feel comfortable discussing death (51.42% versus 29.23%, *p* < 0.001), and support family involvement (95.33%). However, challenges such as discomfort in discussing death (57.29%) and frustration with time demands (57.96%) highlight the need for structured education and policy interventions. To address these gaps, we recommend enhancing EOL training through standardized curricula, integrating EOL care as a core competency in paramedic programs, and conducting further research on its long-term impact. Strengthening paramedics’ preparedness in EOL care is essential to ensuring compassionate, patient-centered, and effective emergency medical services.

## Figures and Tables

**Table 1:** The demographic and work-related characteristics of the included paramedics.

Variable	Number	Percentage (%)
**Age**		
18–25	51	4.86
26–35	571	54.43
36–45	423	40.32
46–55	4	0.38
**Gender**		
Female	361	34.41
Male	688	65.59
**Marital status**		
Single	401	38.41
Married	643	61.59
**Job description**		
EMT	11	1.05
Paramedic	1037	98.95
**Educational level**		
Diploma	14	1.33
Bachelor	1026	97.81
Master	5	0.48
PhD or MD degree	4	0.38
**Work setting**		
Station-based	431	41.09
Hospital-based	618	58.91
**Workplace**		
Private sector	582	55.48
Government sector	467	44.52
**Geographical area**		
Rural	580	55.29
Urban	469	44.71
**Years of experience**		
<1	157	14.97
1–5	253	24.12
6–10	367	34.99
11–15	269	25.64
>15	3	0.29
**Daily working hours**		
6 h	20	1.91
8 h	245	23.36
12 h	520	49.57
24 h	264	25.17
**Number of occasions dealing with EOL patients**		
None	574	54.72
1–3 times	180	17.16
4–6 times	19	1.81
7–10 times	273	26.02
>10 times	3	0.29

**Table 2:** Paramedics’ attitudes toward caring for end-of-life patients.

Variable	Number	Percentage (%)
**End-of-life care is a key role in paramedics’ job.**		
No	361	34.41
Yes	688	65.59
**In the past, did you attend a seminar or a lecture on palliative care?**		
No	575	54.81
Yes	474	45.19
**Did you participate in any end-of-life care courses in the past?**		
No	520	49.57
Yes	529	50.43
**During your undergraduate studies, was end-of-life care included in your curriculum?**		
No	30	2.86
Yes	1019	97.14
**Paramedics are familiar with their duties in end-of-life care.**		
Disagree	4	0.38
Agree	994	94.76
Unsure	51	4.86
**Caring for a dying patient is a worthwhile experience.**		
Disagree	19	1.81
Agree	1001	95.42
Unsure	29	2.76
**I feel uncomfortable talking about impending death with dying patients.**		
Disagree	424	40.42
Agree	601	57.29
Unsure	24	2.29
**The length of time spent giving care to a dying patient frustrates me.**		
Disagree	418	39.85
Agree	608	57.96
Unsure	23	2.19
**A dying patient’s family should be involved in his or her physical care.**		
Disagree	18	1.72
Agree	1000	95.33
Unsure	31	2.96
**I hope I am not present when an end-of-life patient is experiencing death.**		
Disagree	29	2.76
Agree	592	56.43
Unsure	428	40.8
**I am afraid of becoming friends with an end-of-life patient.**		
Disagree	417	39.75
Agree	606	57.77
Unsure	26	2.48
**Families need emotional support to accept the behavioral changes of an end-of-life patient.**		
Disagree	13	1.24
Agree	1010	96.28
Unsure	26	2.48
**Paramedics are qualified in determining the proper intervention while taking care of end-of-life patients.**		
Disagree	16	1.53
Agree	1007	96
Unsure	26	2.48
**End-of-life patients should be given honest answers about their conditions.**		
Disagree	23	2.19
Agree	449	42.8
Unsure	577	55
**Family members who stay close to an end-of-life patient often interfere with paramedics’ work during caring for the patient.**		
Disagree	11	1.05
Agree	1008	96.09
Unsure	30	2.86
**As a paramedic, I would like to work in end-of-life care.**		
Disagree	14	1.33
Agree	1005	95.81
Unsure	30	2.86

**Table 3:** Paramedics’ demographic and work characteristics, stratified by receiving prior EOL courses.

Variable	Received EOL Course		*p* Value
	No (N = 520)	Yes (N = 529)	
**Age**			
18–25	10 (1.92)	41 (7.75)	<0.001
26–35	356 (68.46)	215 (40.64)	
36–45	152 (29.30)	271 (51.22)	
46–55	2 (0.38)	2 (0.38)	
**Gender**			
Female	355 (68.26)	6 (1.13)	<0.001
Male	165 (31.73)	523 (98.87)	
**Marital status**			
Single	360 (69.23)	41 (7.75)	<0.001
Married	159 (30.57)	484 (92.25)	
**Job description**			
EMT	3 (0.57)	8 (1.51)	0.136 *
Paramedic	517 (99.43)	520 (98.49)	
**Educational level**			
Diploma	5 (0.96)	9 (1.7)	0.076 *
Bachelor’s	514 (98.84)	512 (96.78)	
Master’s	1 (0.19)	4 (0.75)	
PhD or MD	0 (0)	4 (0.75)	
**Work setting**			
Station-based	156 (30)	275 (51.99)	<0.001
Hospital-based	364 (70)	254 (48.01)	
**Workplace**			
Private sector	354 (68.07)	228 (43.11)	<0.001
Government sector	166 (31.93)	301 (56.89)	
**Geographical area**			
Rural	357 (68.65)	223 (42.15)	<0.001
Urban	163 (31.35)	306 (57.85)	
**Years of experience**			
<1	154 (29.61)	3 (0.57)	<0.001 *
1–5	8 (1.53)	245 (46.14)	
6–10	355 (68.26)	12 (2.27)	
11–15	1 (0.19)	268 (50.66)	
>15	2 (0.38)	1 (0.19)	
**Daily working hours**			
6 h	6 (1.15)	14 (2.64)	<0.001 *
8 h	7 (1.35)	238 (44.99)	
12 h	507 (97.5)	13 (2.45)	
24 h	0 (0)	264 (49.91)	
**Number of occasions dealing with EOL patients**			
None	354 (68.08)	220 (41.58)	<0.001 *
1–3 times	158 (30.38)	22 (4.16)	
4–6 times	3 (0.57)	16 (3.02)	
7–10 times	4 (0.77)	269 (50.85)	
>10 times	1 (0.19)	2 (0.38)	

* Fischer’s exact test was used instead of the chi-squared test. Data are presented as numbers (percentages).

**Table 4:** Paramedics’ attitudes toward caring for end-of-life patients, stratified by receiving prior EOL courses.

Variable	Received EOL Course		*p* Value
	No (N = 520)	Yes (N = 529)	
**End-of-life care is a key role in paramedics’ job.**			
No	353 (67.88)	8 (1.51)	<0.001
Yes	167 (32.12)	521 (98.49)	
**In the past, did you attend a seminar or a lecture on palliative care?**			
No	359 (69.04)	216 (48.83)	<0.001
Yes	161 (30.96)	313 (59.17)	
**During your undergraduate studies, was end-of-life care included in your curriculum?**			
No	11 (2.12)	19 (3.59)	0.151
Yes	509 (97.88)	510 (96.41)	
**Paramedics are familiar with their duties in end-of-life care.**			
Disagree	2 (0.38)	2 (0.38)	<0.001 *
Agree	510 (98.08)	484 (91.49)	
Unsure	8 (1.54)	43 (8.12)	
**Caring for a dying patient is a worthwhile experience.**			
Disagree	3 (0.58)	16 (3.02)	<0.001 *
Agree	511 (98.27)	490 (92.62)	
Unsure	6 (1.15)	23 (4.35)	
**I feel uncomfortable talking about impending death with dying patients.**			
Disagree	152 (29.23)	272 (51.42)	<0.001
Agree	361 (69.42)	240 (45.37)	
Unsure	7 (1.35)	17 (3.21)	
**The length of time spent in giving care to a dying patient frustrates me.**			
Disagree	151 (29.04)	267 (50.47)	<0.001
Agree	360 (69.23)	248 (46.88)	
Unsure	9 (1.73)	14 (2.65)	
**A dying patient’s family should be involved in his or her physical care.**			
Disagree	4 (0.77)	14 (2.65)	<0.001 *
Agree	511 (98.27)	489 (92.44)	
Unsure	5 (0.96)	26 (4.91)	
**I hope I am not present when an end-of-life patient is experiencing death.**			
Disagree	2 (0.38)	27 (5.1)	<0.001 *
Agree	362 (69.62)	230 (43.48)	
Unsure	156 (30)	272 (51.42)	
**I am afraid of becoming friends with an end-of-life patient.**			
Disagree	152 (29.23)	265 (50.09)	<0.001 *
Agree	364 (70)	242 (45.74)	
Unsure	4 (0.77)	22 (4.16)	
**Families need emotional support to accept the behavioral changes of an end-of-life patient.**			
Disagree	2 (0.38)	11 (2.08)	<0.001 *
Agree	513 (98.65)	497 (93.95)	
Unsure	5 (0.96)	21 (3.97)	
**Paramedics are qualified in determining the proper intervention while taking care of end-of-life patients.**			
Disagree	3 (0.58)	13 (2.46)	0.003 *
Agree	510 (98.08)	497 (93.95)	
Unsure	7 (1.35)	19 (3.59)	
**End-of-life patients should be given honest answers about their conditions.**			
Disagree	8 (1.54)	15 (2.834)	<0.001
Agree	159 (30.58)	290 (54.82)	
Unsure	353 (67.88)	224 (42.34)	
**Family members who stay close to an end-of-life patient often interfere with paramedics’ work during caring for the patient.**			
Disagree	3 (0.58)	8 (1.51)	0.004 *
Agree	510 (98.65)	498 (94.14)	
Unsure	7 (1.35)	23 (43.47)	
**As a paramedic, I would like to work in end-of-life care.**			
Disagree	5 (0.96)	9 (1.7)	0.021
Agree	507 (97.5)	498 (94.14)	
Unsure	8 (1.54)	22 (4.16)	

* Fischer’s exact test was used instead of the chi-squared test. Data are presented as numbers (percentages).

## Data Availability

The original contributions presented in this study are included in the article. Further inquiries can be directed toward the corresponding authors.
